# Sensitivity and responses of chloroplasts to salt stress in plants

**DOI:** 10.3389/fpls.2024.1374086

**Published:** 2024-04-17

**Authors:** Xuemei Wang, Zengting Chen, Na Sui

**Affiliations:** Shandong Provincial Key Laboratory of Plant Stress, College of Life Sciences, Shandong Normal University, Jinan, China

**Keywords:** salt stress, chloroplast, photosynthesis, ROS, ion transport, response

## Abstract

Chloroplast, the site for photosynthesis and various biochemical reactions, is subject to many environmental stresses including salt stress, which affects chloroplast structure, photosynthetic processes, osmotic balance, ROS homeostasis, and so on. The maintenance of normal chloroplast function is essential for the survival of plants. Plants have developed different mechanisms to cope with salt-induced toxicity on chloroplasts to ensure the normal function of chloroplasts. The salt tolerance mechanism is complex and varies with plant species, so many aspects of these mechanisms are not entirely clear yet. In this review, we explore the effect of salinity on chloroplast structure and function, and discuss the adaptive mechanisms by which chloroplasts respond to salt stress. Understanding the sensitivity and responses of chloroplasts to salt stress will help us understand the important role of chloroplasts in plant salt stress adaptation and lay the foundation for enhancing plant salt tolerance.

## Introduction

As sessile organisms, plants are subjected to various environmental stresses, including salt stress. Salinity has become one of the most severe environmental stresses that limit plant growth and development as well as crop yield (Munns and Tester, 2008; Acosta-Motos et al., 2017). It is estimated that approximately more than 800 million hectares of arable lands are affected by salinity worldwide, accounting for over 6% of the total lands worldwide (Munns and Tester, 2008; Flowers et al., 2010; Liu and Wang, 2021). If soil salinization continues, 50% of cultivable lands will be salinized by 2050 (Hasanuzzaman et al., 2014; Hossain, 2019), resulting in a reduction of arable lands, which will inevitably reduce crop productivity. Salinity affects most physiological and biochemical processes, including photosynthesis, the biosynthesis of amino acid, lipid metabolism, protein synthesis, and RNA metabolism (Munns and Tester, 2008; Yan et al., 2013; Abdelhamid et al., 2020; Hameed et al., 2021). As the primary sites for photosynthesis and other metabolic processes, chloroplasts are also damaged by salt stress.

Chloroplasts are specialized plastids found in plants and algae, and their main function is photosynthesis, through which chloroplasts produce energy for plant growth and crop yield. In addition to photosynthesis, chloroplasts also carry out a variety of other important roles, such as the biosynthesis of amino acids, fatty acids, nucleotides, lipids, vitamins, phytohormones, the manufacture of starch and pigments, as well as the reduction of sulfates and nitrites (Neuhaus and Emes, 2000; van Wijk, 2000; Suo et al., 2017; Li et al., 2022). Thus, any perturbation of chloroplast function will impair plant growth and development, as well as crop yield.

**Figure 1:**
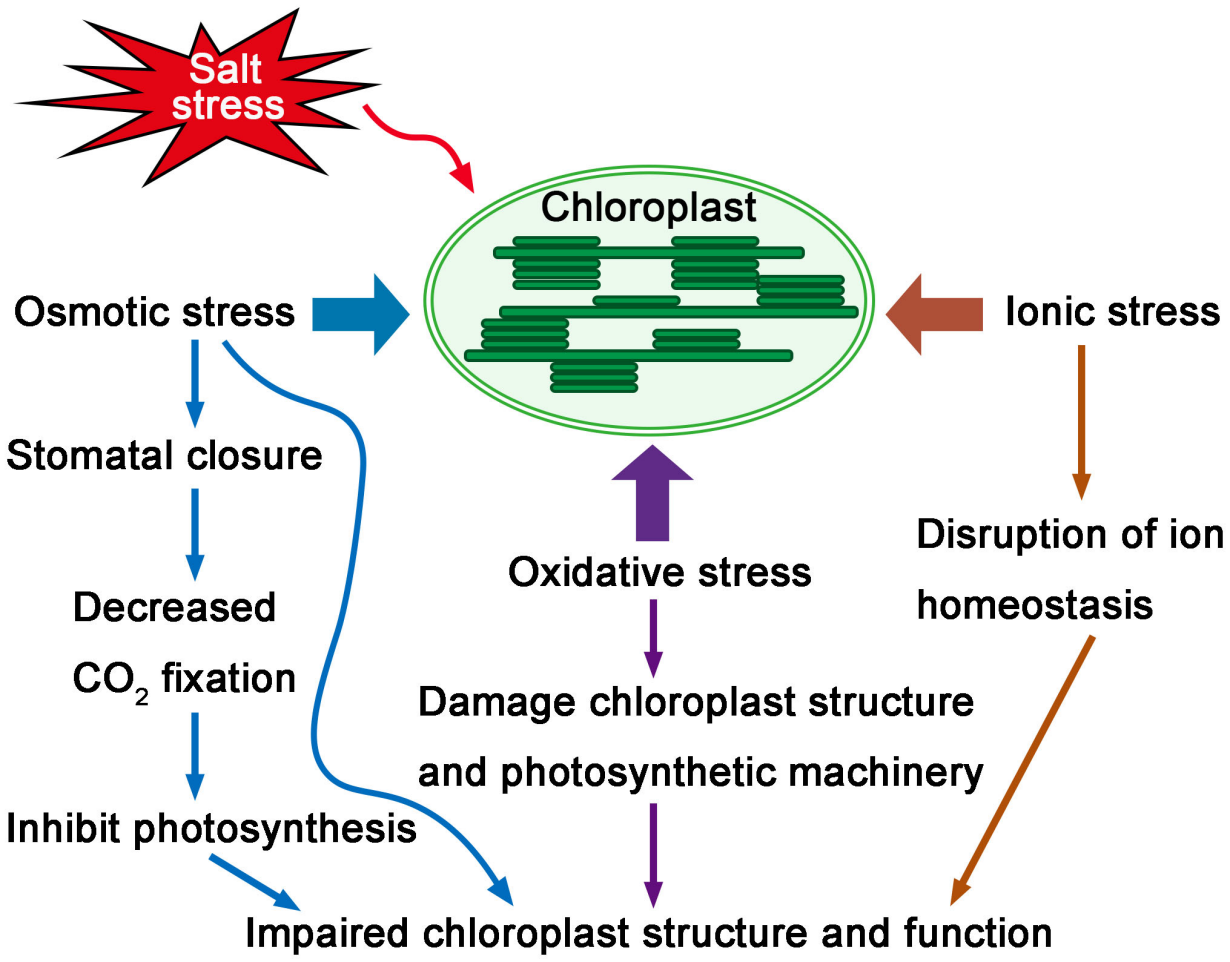
Sensitivity of chloroplasts under salt stress. Salt stress causes osmotic stress, ionic stress and oxidative stress to chloroplasts. The initial osmotic stress causes stomatal closure, which in turn affects the CO_2_ fixation of photosynthesis. Excess accumulation of Na^+^ also disrupts ion homeostasis inside chloroplasts. Salt stress causes excess ROS accumulation, which leads to oxidative stress to chloroplasts and damages chloroplast structure and photosynthetic machinery. Thus, salt stress negatively affects chloroplast structure and function.

Chloroplasts are highly sensitive to salt stress (Nouri et al., 2015). Photosynthesis, as the major function of chloroplasts, is affected by salt stress. The initial impact of salinity on plants is osmotic stress, which causes stomatal closure (Akyol et al., 2020; Ben Amor et al., 2020). Stomatal closure in turn affects the carbon fixation in photosynthesis by limiting CO_2_ supply (Chaves et al., 2009; Yan et al., 2013; Singh and Roychoudhury, 2021). In addition to CO_2_ fixation, photoreaction processes are also affected by salt stress due to many non-stomatal limitations, including changed activities of CO_2_ fixing enzymes (Delatorre-Herrera et al., 2021), damaged photosynthetic apparatus (Maxwell and Johnson, 2000; Huang et al., 2019), a reduction in photosynthetic pigments (Demetriou et al., 2007), and the inhibition of electron transport from PSII to PSI (He et al., 2021; Zahra et al., 2022). The excess accumulation of Na^+^ and Cl^-^ ions inside chloroplasts not only affects photosynthetic components (Davenport et al., 2005), but also inhibits the uptake of K^+^ and Ca^2+^ ions, which disrupts ion homeostasis (Hu and Schmidhalter, 2005; Bose et al., 2017). In addition, when plants are exposed to salt stress, the decline of carbon assimilation and the reduced photosynthetic electron transport rate will increase reactive oxygen species (ROS) production, that leads to oxidative stress (Munns and Tester, 2008; Huang et al., 2019; Lu et al., 2023). Thus, salt stress induces osmotic stress, ionic stress and oxidative stress to chloroplasts and negatively affects the function of chloroplasts (**Figure 1**).

Chloroplasts have evolved sophisticated mechanisms to acclimate to salt stress. For example, the xanthophyll cycle participates in dissipating excess excitation energy in PSII. Ascorbate and water-water cycle can protect photosynthetic machinery from oxidative damage (Acosta-Motos et al., 2017). Chloroplasts possess candidate Na^+^, K^+^, Cl^-^ ion transporters that can regulate ion concentrations, however the ion transport capacity of chloroplasts differs between salt-tolerant and salt-sensitive plants (Bose et al., 2017). In response to osmotic stress under salinity, plant cells not only uptake inorganic ions from the external environment (Zhao et al., 2002; Cui et al., 2007; Huang et al., 2019), but also produce organic osmolytes such as sorbitol, mannitol, proline, glycine betain, polyamines, etc., most of which are localized in the chloroplast (Shen et al., 1997a, Shen et al., 1997b; Hameed et al., 2021). Chloroplasts are major ROS production sites, and salinity increases the ROS accumulation, causing oxidative stress to chloroplasts. Certain enzymatic and non-enzymatic antioxidants are present in chloroplasts to scavenge ROS (Mittler et al., 2004). Tightly regulated levels of ROS can also function as retrograde signals for chloroplasts to communicate with the nucleus through retrograde signaling (Uzilday et al., 2014; Hameed et al., 2021).

In this review, we summarize the effects of salt stress on chloroplast structure and cellular processes occurring within them. We also discuss the mechanisms through which chloroplasts respond and adapt to salt stress. Together, these studies suggest the significant roles of chloroplasts in plant adaptation to salt stress. Furthermore, studies on the adaptive mechanisms of chloroplasts in response to salt stress will contribute to improving crop yield under salt stress, and the genes that have been validated to be important for chloroplast function are also valuable for developing salt-tolerant crops through genetic engineering.

## Chloroplast sensitivity to salt stress

### Salt sensitivity of chloroplast ultrastructure

Chloroplasts are sensitive to salt stress, which affects chloroplast size, number, lamellar organization, starch accumulation and so on (Zahra et al., 2022). Under normal conditions, chloroplasts have ellipsoidal shapes, with regularly organized grana stacks and dense stromal thylakoid lamellae (Zhang et al., 2020). While salt stress makes chloroplasts deform into irregular shapes, with reduced grana stacks (Shu et al., 2015). Excessive salinity also led to thylakoid swelling in the chloroplasts of *Thellungiella salsuginea*, which was primarily caused by disruption of chloroplast osmotic equilibrium (Goussi et al., 2018). Thus, high salinity changed chloroplast shapes and lamellar organization.

In many studies, larger starch granules under salt stress have been reported (Fidalgo et al., 2004; Barhoumi et al., 2007; Štefanić et al., 2013; Goussi et al., 2018; Manaa et al., 2019). For instance, Štefanić et al. (2013) reported that salt stress caused starch accumulation in the chloroplast of wheat cultivars, resulting from the decreased sucrose biosynthesis and increased starch biosynthesis. The starch accumulation may also be related to the ionic composition changes of starch-degrading enzymes (Salama et al., 1994). The accumulation of starch granules in chloroplasts under salt stress is thought to play important roles as osmolytes to help chloroplasts absorb water and maintain structural integrity, while also providing energy for cells (Queirós et al., 2011; Chang et al., 2015). Notably, studies have found that the starch granules ultimately degrade under high salinity or prolonged salt stress, possibly attributed to the large amount of energy that plants require to sustain life (Gao et al., 2015; Huang et al., 2019; Alkhatib et al., 2021; Vineeth et al., 2023). In addition, the accumulation of plastoglobules under salt stress has also been reported in many cases (Evelin et al., 2013; Barkla et al., 2018; Manaa et al., 2019). Plastoglobules are sites for the synthesis of tocopherols under oxidative stress, and an increased number of plastoglobules can prevent salt-induced oxidative damage to the thylakoid membrane (Evelin et al., 2013). Thus, the accumulation and degradation of starch granules as well as the accumulation of plastoglobules are for chloroplasts to adapt to salt stress.

Under salt stress, the degree of damage to chloroplasts varies from plant to plant differing in their ability to cope with salinity. For instance, chloroplasts of spinach (salt-tolerant) decreased in volume in the presence of salt (Delfine et al., 1998), while chloroplasts of Arabidopsis (salt-sensitive) showed an increase in volume under salinity (Štefanić et al., 2013). Changes in chloroplast volume may be attributed to alterations in stromal ionic composition (Hameed et al., 2021). Bose et al. (2017) reported that salt entry into the chloroplast stroma may be crucial for grana formation in halophytes, whose chloroplasts use Na^+^ in functional roles.

### Salt sensitivity of photosynthesis

The most important physiological process occurring in chloroplasts is photosynthesis, which is the energy source for plant growth and development and plays crucial roles in plant productivity (Yang et al., 2020; Zahra et al., 2022; Lu et al., 2023). Photosynthesis comprises light reaction and dark reaction phases. The light reaction process involves the primary reaction and photosynthetic electron transport (PET), through which the light energy is converted into active chemical energy in the form of reducing power and ATP (Arnon, 1959; Bowyer and Leegood, 1997). The dark reaction is the process of photosynthetic carbon fixation, which reduces carbon dioxide to carbohydrates under the catalysis of various enzymes (Nouri et al., 2015).

Photosynthesis is sensitive to salt stress. Photosynthetic activity and photosynthetic rate, which are important factors affecting plant productivity and crop yield, have been found to be decreased in many plants under salt stress (Parida and Das, 2005; Hiyane et al., 2010; Yan et al., 2013; MaChado and Serralheiro, 2017; Lekklar et al., 2019). The decrease of photosynthetic activity and photosynthetic rate was caused by various reasons. The initial effect of salt stress on photosynthesis is the decreased CO_2_ availability caused by stomatal limitation, resulting from elevated osmotic pressure and leading to a decrease in carbon fixation (Flexas et al., 2004; Parida and Das, 2005; Chaves et al., 2009; Yan et al., 2013).

Besides CO_2_ fixation, photoreaction is also sensitive to salt stress (Tseng et al., 2007; Flexas et al., 2012; Munns et al., 2019). The negative effect of salt stress on photoreaction is mainly reflected in electron transport chain (ETC) and photosystems (Huang et al., 2019). High salinity blocks electron transport chain, resulting in excessive accumulation of ROS which cause oxidative damage to thylakoid membrane proteins, lipids, membranes, and photosynthetic enzymes (Lu et al., 2023). The inhibition of electron transport and the oxidative damage to photosynthetic apparatus under salt stress resulted in photoinhibition to photosystems. PSII is considered to be more susceptible to photoinhibition. Salt stress blocks PSII-mediated electron transport, resulting in accumulation of electrons available for charge separation that are prone to induce PSII photoinhibition with excess ROS production (Murata et al., 2007; Ahanger and Agarwal, 2017). The accumulation of ROS inhibits the repair of PSII through suppression of the *de novo* synthesis of PSII proteins, especially of the D1 protein, causing inactivation of PSII (Nishiyama et al., 2001; Allakhverdiev et al., 2002; Takahashi and Murata, 2008). Photoinhibition impacts the efficiency of photosystems. Under saline conditions, a general decrease in the maximal photochemical efficiency of PSII (*Fv/Fm*) occurs. For instance, under salt stress, the *Fv/Fm* declined in *P. granatum* compared to the control (Khayyat et al., 2014). The decrease in *Fv/Fm* was also reported in olives, which probably indicates photodamage under salt stress (Loreto et al., 2003). Compared to PSII, PSI is less sensitive to salt stress, but repairing the damaged PSI is more difficult, thus, PSI photoinhibition is more harmful (He et al., 2021).

CO_2_ fixation processes are generally considered to be more sensitive to salt stress than photoreaction processes (Gulzar et al., 2020). Besides stomatal limitation, photosynthetic enzymes also play significant roles in CO_2_ assimilation and are affected by salinity. Ribulose-1,5-bisphosphate carboxylase/oxygenase (Rubisco) is a crucial photosynthetic enzyme catalyzing CO_2_ fixation during the C_3_ pathway, and it has been reported that salt stress inhibits Rubisco activity in most species (Hameed et al., 2021). However, increased Rubisco activity under salt stress was also observed in *Kalidium foliatum* (Gong et al., 2018). Rubisco activase (RCA) is important for Rubisco activity. Salt stress enhanced RCA activity in many plant species, such as alfalfa (Xiong et al., 2017) and *S.salsa* (Li et al., 2011). Enhanced RCA activity can help activate the available Rubisco. Phosphoenolpyruvate carboxylase (PEPC) is a crucial photosynthetic enzyme catalyzing CO_2_ fixation during the C_4_ pathway. Under salt stress, an increase in PEPC activity was reported in C_4_ species maize (dos Santos Araújo et al., 2021) and *Bienertia sinuspersici* (Leisner et al., 2010). Increased PEPC activity can help enhance CO_2_ fixation for C_4_ plants when atmospherical CO_2_ concentration is low or during stomatal limitation (Huang et al., 2019; Hameed et al., 2021). In fact, the effect of salinity on these enzymes varies among plants differing in their salt tolerance, and further studies are needed to explore the relationship between these enzymes and plant salt tolerance.

### Effects of salt stress on ion homeostasis inside chloroplasts

Chloroplasts contain three types of membranes: the outer and inner envelope membranes, and the thylakoid membrane. Ion channels and transporters are located in these membranes to transport ions in and out of them, however, our current knowledge on specific chloroplast ion transporters that affect ion concentrations under salt stress is limited (Hameed et al., 2021).

Salt stress causes increases in Na^+^ and Cl^-^ concentrations and decreases in K^+^ concentration inside chloroplasts (Robinson and Downton, 1984, Robinson and Downton, 1985), resulting in ion stress and osmotic stress (Munns and Tester, 2008).

Regulating ion (K^+^, Na^+^, Cl^-^) homeostasis is crucial for maintaining chloroplast function. Under low salt conditions, halophytes accumulate about 20-fold higher Na^+^ inside chloroplasts than glycophytes (Robinson and Downton, 1984, Robinson and Downton, 1985). Halophytes use Na^+^ in functional roles (Bose et al., 2017). The lack of Na^+^ induced chlorosis in *Atriplex vesicaria* (Brownell and Wood, 1957; Brownell, 1965), and reduced PSII activity in *Amaranthus tricolor* and *Kochia childsii* (Grof et al., 1989; Johnston et al., 1989). Furthermore, when the Na^+^ concentration of the growth medium exceeds 300 mM, but halophyte chloroplast Na^+^ concentrations are still kept much lower, and halophytes can complete their life cycle. In contrast, glycophytes cannot tolerate so high salt concentration (Bose et al., 2017). Thus, it is assumed that halophyte chloroplasts have Na^+^ transporters to regulate Na^+^ concentrations in the optimal range. K^+^ promotes the chlorophyll synthesis and Rubisco synthesis, and is necessary for chloroplast development, ultrastructure, and volume regulation. Maintaining optimal K^+^/Na^+^ ratio within chloroplasts is critical to photosynthesis (Bose et al., 2017). K^+^ deficiency damages chloroplast ultrastructure, for instance, the irregular lamella structure and reduced numbers of grana and lamellae were observed in maize under K^+^ deficiency stress (Du et al., 2019). It has also been reported that K^+^ deficiency reduces the concentration of chlorophyll and the activity and quantity of Rubisco (Tränkner et al., 2018). Salt stress causes K^+^ loss from chloroplasts of both halophytes and glycophytes (Robinson and Downton, 1984, Robinson and Downton, 1985). Cl^-^ is a cofactor of PSII complex, and it also contributes to stabilizing chloroplast membrane potentials, thus, Cl^-^ is crucial for photosynthesis (Herdean et al., 2016a). It has been reported that the excess accumulation of Cl^-^ within chloroplasts affects photosynthesis of many crop species (Teakle and Tyerman, 2010). However, in other instances, high concentrations of Cl^-^ inside chloroplasts contribute to increasing electron transport in some halophytes under salt stress (Critchley, 1982). It has been considered that halophytes have mechanisms to maintain steady Cl^-^ concentrations and regulate Cl^-^ homeostasis within chloroplasts. Therefore, halophyte chloroplasts use Na^+^, K^+^, Cl^-^ ions in functional roles and have more efficient transport mechanisms to maintain ion homeostasis than glycophyte chloroplasts.

### Effects of salt stress on chloroplast ROS generation

Chloroplasts are major sites for ROS production in plants subjected to environmental stresses, such as salt stress. In chloroplasts, the triplet chlorophylls and the photosynthetic electron transport chains in PSII and PSI, all produce ROS (Edreva, 2005). The reduction of CO_2_ assimilation rates under salt stress results in the over-reduction of PSII along with electrons transporting to molecular oxygen, which generates ROS (Bose et al., 2013; Ozgur et al., 2013; Gulzar et al., 2020). Under salt stress, electrons are prone to be transported from Ferredoxin to molecular oxygen, rather than NADP^+^, the final electron acceptor in PSI, and this pathway produces O_2_^.-^, which is dismutated into H_2_O_2_ and O_2_ by SOD (Asada, 1999). Salt stress causes a decrease in photosynthetic activity of plants and excess protons cannot be utilized, which leads to photoinhibition of PSII and the production of ROS (Hideg et al., 2002). In addition, ROS can be produced in chloroplasts through other ways, for instance, singlet oxygen can be generated within chloroplasts by energy transfer from the excited chlorophyll (^1^chl) to the ground state chlorophyll (chl) (Liu et al., 2021).

There are differences in ROS levels between the chloroplasts of salt-tolerant and salt-sensitive plants. An increase in H_2_O_2_ levels was reported in chloroplasts of two pea cultivars under salt stress, moreover, the salt-sensitive cultivar produced much higher H_2_O_2_ levels than the salt-tolerant one (Hernández et al., 1995; Acosta-Motos et al., 2017). A substantially higher H_2_O_2_ level was observed in chloroplasts of a halophyte *T. salsuginea* than glycophyte *Arabidopsis thaliana* under normal growth conditions. However, under salt stress, *Arabidopsis* produced a higher H_2_O_2_ level than *T. salsuginea* (Wiciarz et al., 2015). The similar results were observed in chloroplasts of two tomato cultivars with different salt tolerance (Ben Hamed et al., 2020). The higher H_2_O_2_ levels in halophytes under stress-free conditions may keep antioxidant system in the activated state, and serve as a signal for adaptive responses, and this phenomenon may indicate that the halophytes are pre-adapted to stress. The mechanism of high H_2_O_2_ levels in halophytes in stress-free conditions, however, still remains unclear. In contrast, salt stress increased H_2_O_2_ levels in both species, but it was significant only in glycophtes, indicating that salt-tolerant plants possess more efficient mechanisms to regulate ROS homeostasis than salt-sensitive plants.

ROS functions as a double-edged sword, tightly controlled levels of ROS can act as stress signaling molecules to trigger specific protective responses (Mittler et al., 2004; Suzuki et al., 2012; Lodeyro et al., 2016). However, if excess ROS accumulates in chloroplasts, it can cause oxidative damage to chloroplasts and reduce the photosynthetic efficiency of plants (Zheng et al., 2017).

## Chloroplast adaptations to salt stress

### Mechanisms for the protection of photosynthetic machinery under salt stress

**Figure 2:**
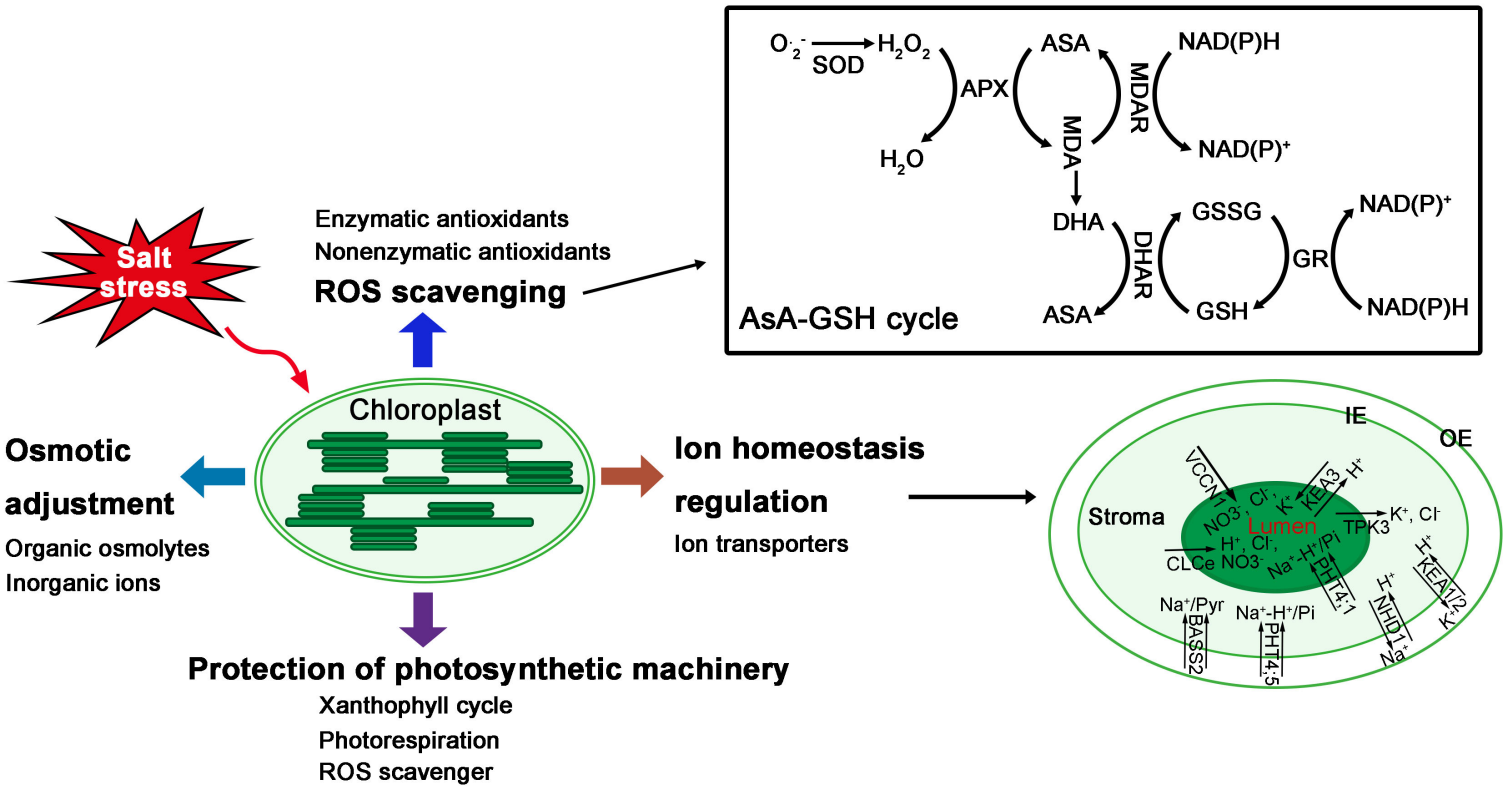
Responses of chloroplasts to salt stress. In response to salt stress, chloroplasts have evolved several mechanisms, such as protection of photosynthetic machinery, ion homeostasis regulation, osmotic adjustment, ROS scavenging, and so on.

Under salt stress, the stomatal limitation and the decrease of ETC lead to the accumulation of excess excitation energy in PSII and the generation of ROS, which can damage the photosynthetic machinery. Plants have developed mechanisms to protect photosystems (Acosta-Motos et al., 2017) (**Figure 2**). Xanthophyll cycle is one of the mechanisms to dissipate excess excitation energy of PSII in the form of heat by non-photochemical quenching (NPQ), potentially preventing the production of ROS (Qiu et al., 2003; Hameed et al., 2021). The violaxanthin de-epoxidase in the xanthophyll cycle consumes NADPH, whose accumulation may cause the over-reduction of PSII, thereby increasing ROS production (Niyogi, 1999; Hameed et al., 2021). Zeaxanthin has been reported to play a role in protecting photosynthetic machinery, since it acts as an antioxidant that scavenges ROS in the thylakoid membranes and relieves salt-induced photo-oxidation (Zhang et al., 2012). In addition, ascorbate has also been reported to play a role in protecting photosynthetic machinery under salt stress (Huang et al., 2005), since ascorbate not only functions as a ROS scavenger, but also acts as a cofactor of violaxanthin de-epoxidase, contributing to the dissipation of excess light energy (Demmig-Adams and Adams, 1992; Acosta-Motos et al., 2017).

Photorespiration is another mechanism for plants to protect photosynthetic machinery. Under salt stress, stomatal limitation makes it difficult for external CO_2_ to enter plant cells, leading to the over-reduction of ETC. Rubisco operates as an oxygenase at this time, and photorespiration is initiated to consume excess reducing power (Huang et al., 2019). The photorespiration pathway produces phosphoglycerate, which enters the Calvin-Benson cycle to regenerate RuBP (Hodges et al., 2016). The photorespiration pathway also produces recycled CO_2_, which is important for CAM plants and C4 plants (Raghavendra and Padmasree, 2003). Photorespiration not only provides substrates for the chloroplast, but also consumes ATP, contributing to the dissipation of excess excitation energy and decreasing the risk of photoinhibition under salt stress (Hodges et al., 2016).

In addition, plant chloroplasts generate ROS during photosynthesis under stresses. The cysteine residues of photosynthetic proteins are susceptible to redox modifications, which can influence protein structure and function, and further influence photosynthesis (Yu et al., 2020). Through redox proteomics, lots of redox-responsive proteins were identified under stresses. For instance, redox proteomics has revealed that redox modified photosystem proteins, soluble electron carriers, ATP synthase, enzymes in the Calvin-Benson cycle, and enzymes in chlorophyll biosynthesis in response to various stresses. The light and dark-dependent redox regulation of electron carriers ensures efficient energy utilization. Redox regulation of chlorophyll biosynthesis contributes to preventing oxidative damage (Yu et al., 2020). Therefore, redox modification plays important roles in photosynthesis.

The accumulation of ROS brings oxidative damage to photosynthetic machinery, thus, scavenging ROS will relieve the oxidative damage, other means to scavenge ROS will be discussed below.

### Ion transporters across chloroplast membranes

Maintenance of chloroplast ion homeostasis is crucial for plant growth. Halophytes are thought to possess transporters to maintain ion homeostasis in chloroplasts (**Figure 2**). Through analyzing online data and proteomic data verification, several nucleus-encoded chloroplast proteins were identified to be candidate ion transporters that may mediate Na^+^, K^+^, and Cl^-^ transport through chloroplast membranes (Kleffmann et al., 2004; Ferro et al., 2010; Hooper et al., 2014; Bose et al., 2017). However, the transport function and their role during salt stress of most of these ion transporters still need to be verified.

The entry of Na^+^ into the chloroplast can be mediated by an inner envelope membrane-localized Na^+^-dependent pyruvate transporter (BASS2), which is more abundant in halophytes than in glycophytes (Furumoto et al., 2011). Zhao et al. (2016) reported that overexpression of *TaBASS2* isolated from a salt-tolerant wheat cultivar could enhance salt tolerance of wheat and *Arabidopsis*. The thylakoid membrane localized inorganic phosphate transporters PHT4;1 and inner envelope localized PHT4;4, and PHT4;5 can mediate Na^+^ - and/or H^+^ -dependent Pi transport (Finazzi et al., 2015), thus changing Na^+^ concentrations inside chloroplasts. The chloroplast envelope membrane localized Na^+^/H^+^ antiporter (NhaD; hereafter NHD)-type transporters have long been reported to mediate Na^+^ efflux from the stroma (Furumoto et al., 2011). In *Arabidopsis*, NHD1 mediates Na^+^ export from chloroplast (Müller et al., 2014). In contrast, in *Mesembryanthemum crystallinum* (a halophyte), salt stress increased *NHD1* transcript levels and caused Na^+^ accumulation in chloroplasts, which suggests that NHD1 may function as a Na^+^ importer (Cosentino et al., 2010). Such opposite function of NHD1 in Na^+^ transport mechanism in different species requires further verification.

In *Arabidopsis*, two inner envelope membrane-localized K^+^ efflux antiporters (KEA1 and KEA2) are involved in mediating K^+^ export out of the stoma in exchange for H^+^ influx (Kunza et al., 2014). The two-pore K^+^ channel TPK3 has also been suggested to function in K^+^ export out of the lumen (Carraretto et al., 2013). In contrast, the thylakoid membrane localized KEA3 has been shown to be involved in importing K^+^ into the lumen in exchange for H^+^ (Armbruster et al., 2014; Kunza et al., 2014), as well as increasing PSII quantum efficiency and CO_2_ fixation under fluctuating light conditions (Armbruster et al., 2014). Yet, it is unknown how KEA3 functions during salt stress.

In *Arabidopsis*, the thylakoid membrane-localized CLCe, belonging to the Cl^-^ channel CLC family, was suggested to function as a Cl^-^ channel participating in Cl^-^ homeostasis after transfer from light to dark (Herdean et al., 2016a). The loss-of-function *clce* mutant showed altered photosynthetic electron transport (Herdean et al., 2016a). The function of CLCe in maintaining Cl^-^ homeostasis during salt stress remains to be established. A thylakoid membrane-localized bestrophin-like protein from *Arabidopsis* was reported to function as a voltage-dependent Cl^-^ channel (AtVCCN1), mediating anion influx into the lumen to fine-tune proton motif force, and adjusting photosynthesis to variable light (Herdean et al., 2016b). The function of AtVCCN1 during salt stress remains to be established.

### Osmoprotectants in chloroplasts

To resist salinity-induced osmotic stress, plants synthesize and accumulate organic osmoprotectants to improve osmolality and cellular water retention capacity. Major organic osmoprotectants include glycine betaine (GB), proline, sugar alcohols/polyols (e.g., sorbitol and mannitol), and polyamines (Suo et al., 2017). These organic osmolytes, also called compatible solutes, have long been considered not to have a negative influence on cellular enzymes and functions (Vineeth et al., 2023). The accumulation of these organic osmolytes has been considered an adaptive response to abiotic stresses.

Among the organic osmolytes, GB and proline are considered the most important solutes during salt stress. They not only function in osmotic adjustment, but also protect photosynthetic apparatus (Vineeth et al., 2023). GB is synthesized in chloroplasts (Park et al., 2007), which can stabilize the protein complexes and protect the integrity of membranes, and accelerate the PSII repair under salt stress, thereby improving photosynthesis and salt tolerance (Tian et al., 2017; Ahmad et al., 2018). Increased GB concentration was observed in many salt-stressed plants, such as sorghum (*Sorghum bicolor*) (Yang et al., 2003), spinach, and barley (Papageorgiou and Murata, 1995). In plants, proline may be synthesized in the chloroplast, cytoplasm, or mitochondria (Szabados and Savouré, 2010; Planchet et al., 2014). In addition to functioning as an osmolyte, proline also contributes to stabilizing membranes and proteins, ROS scavenging, and cellular redox homeostasis under salt stress (Ashraf and Foolad, 2007). Similar to GB, salt stress also increased proline accumulation in many plants, such as rice (Demiral and Türkan, 2004; Acosta-Motos et al., 2017). Besides, exogenous application of proline to rice plants improved their salt tolerance (Wutipraditkul et al., 2015). Sugar alcohols, such as mannitol, sorbitol, and trehalose, regulate K^+^/Na^+^ ratio to a low level and protect membranes under salt stress, thus improving photosynthetic rates (Garg et al., 2002; Hameed et al., 2021).

In fact, the notion of osmotic adjustment through organic osmolytes is still controversial. The main reasons include the high cost of synthesis for organic osmolytes and their lower concentration compared to the inorganic ions (Hameed et al., 2021). Inorganic ions appear to be more cost-effective osmolytes to maintain osmolality. However, excess Na^+^ is toxic and plant cells need to maintain Na^+^/K^+^ balance, also studies showed that the difference of salt tolerance between two rice genotypes was related to the accumulation of organic osmolytes but not to Na^+^, K^+^ concentrations (Li et al., 2017). Thus, it seems that osmotic adjustment is collectively achieved by organic osmolytes and inorganic ions (**Figure 2**).

### ROS scavenging

To cope with oxidative damage caused by ROS accumulation under salt stress, chloroplasts have evolved enzymatic and nonenzymatic scavenging mechanisms (Mittler et al., 2004). The enzymatic scavenging mechanism mainly includes water-water cycle and ascorbate-glutathione (AsA-GSH) cycle, which involve many enzymatic antioxidants, such as superoxide dismutases (SOD), ascorbate peroxidase (APX), monodehydroascorbate reductase (MDHAR), dehydroascorbate reductase (DHAR) and glutathione reductase (GR) (Hameed et al., 2021). Besides, the enzymatic scavenging mechanism also include thioredoxin/peroxiredoxin (Trx/Prx) and glutathione peroxidase (GPX) pathways (Suo et al., 2017). Meanwhile, ascorbate, glutathione, tocopherol, and carotenoids constitute the nonenzymatic antioxidant system in chloroplasts (Mittler et al., 2004).

The water-water cycle is an important ROS scavenging pathway in chloroplasts and plays a crucial role in salinity tolerance (Suo et al., 2017). In this cycle, the thylakoid membrane-attached copper/zinc superoxide dismutase (Cu/Zn SOD) converts O_2_^.-^ produced in ETC into O_2_ and H_2_O_2_, which is then reduced to H_2_O by the thylakoid membrane-bound APX (tAPX) (Edreva, 2005). Many studies have reported that the enzymatic antioxidants are upregulated under salt stress and their transgenic plants show enhanced salinity tolerance compared to wild type plants. For example, overexpression of *Cu/Zn-SOD* in *Arabidopsis* (Wu et al., 2016) and cotton chloroplasts (Luo et al., 2013) increased SOD activity and enhanced the plants′ salinity tolerance. Similarly, tobacco plants with an overexpression of *APX* showed higher APX activity and enhanced salinity tolerance (Badawi et al., 2004). The AsA-GSH cycle is an extension of the water-water cycle in chloroplasts (Hameed et al., 2021), it leads to the full scavenging of H_2_O_2_ at the expense of consuming NADPH. Besides SOD and APX that directly scavenge ROS, this cycle also involves other enzymes such as MDHAR, DHAR, and GR, which act as antioxidant-regenerating enzymes that regenerate GSH and ASA from their oxidized forms (Mittler, 2002; Yan et al., 2013). Overexpression of *MDHAR* and *DHAR* in tobacco plants increased enzyme activities and improved plant survival under salt stress (Kavitha et al., 2010; Le Martret et al., 2011). In rice, the expression of OsGR3 was greatly increased under salt treatment (Wu et al., 2013), and the knockout of *OsGR3* increased the plants′ salinity sensitivity (Wu et al., 2015). The Trx/Prx pathway and GPX pathway primarily scavenge salinity-induced H_2_O_2_ in chloroplasts (Suo et al., 2017). The Trx/Prx system scavenges H_2_O_2_ and is reduced by thioredoxin reductase (TrxR) using NADPH as an electron donor (Sevilla et al., 2015). GPX also reduces H_2_O_2_ using GSH as an electron donor (Zhai et al., 2013). Therefore, these enzymatic scavenging systems in chloroplasts are important for redox homeostasis and contribute to improving plants salinity tolerance.

OH• and ^1^O_2_, which are not targets of the enzymatic scavenging system, can be solely scavenged by nonenzymatic antioxidants, such as AsA, GSH, carotenoids, and tocopherol in chloroplasts (Edreva, 2005; Vineeth et al., 2023). Tocopherol and carotenoids scavenge ^1^O_2_ mainly by the electronic energy transfer mechanism (Khorobrykh et al., 2020). Tocopherol is a thylakoid membrane-localized lipid antioxidant, and carotenoids are components of the thylakoid pigment-protein complexes, both tocopherol and carotenoids are involved in protecting chloroplast thylakoid membrane from oxidative damage (Suo et al., 2017; Liu et al., 2021).

In short, the enzymatic and nonenzymatic scavenging mechanisms in chloroplasts collaborate to help prevent chloroplasts from oxidative damage due to ROS accumulation under salt stress (**Figure 2**), and to maintain ROS levels within a “functionally useful” range for initiating stress responses. Many genes encoding antioxidants have been shown to be helpful for improving salinity tolerance in various plant species, and the ROS scavenging systems in chloroplasts play a vital role in salinity tolerance (Suo et al., 2017).

## Conclusions and future prospects

Salinity is one of the severe abiotic stresses limiting the growth and development of plants, especially crops, and poses a threat to the sustainability of global agriculture. Salt stress impacts the structure and function of the chloroplast, which is the site for photosynthesis and other metabolic reactions, thus chloroplast dysfunction as a result of environmental stresses, including salt stress, will affect plants (Bose et al., 2013). Chloroplasts are sensitive to environmental stresses and act as sensors to sense stress signals and help plants adapt to adverse environments. Understanding the sensitivities and responses of chloroplasts to salt stress is necessary for improving crop production. Increasing studies have revealed the importance of chloroplasts in plant salt stress response and adaptation (Song et al., 2021).

In this review, we have discussed the negative effects of salt stress on the structure and function of chloroplasts, for example, salt stress affects chloroplast size, shape, starch granules, and plastoglobules accumulation. Salt stress also negatively affects photosynthesis including CO_2_ fixation and photoreaction processes, as well as disrupts ion homeostasis and leads to ROS accumulation in chloroplasts. Chloroplasts have evolved fine-tuned pathways to respond to salt stress. For example, xanthophyll cycle, photorespiration, and ascorbate have been reported to play roles in protecting photosynthetic machinery. Ion transporters across chloroplast membranes are involved in ion homeostasis regulation. Several organic osmolytes are synthesized in chloroplasts to function in osmotic adjustment. Chloroplasts also possess enzymatic and nonenzymatic scavenging systems to scavenge ROS. In addition, proteomics studies under salt stress have revealed lots of chloroplast proteins playing critical roles in photosystems, Calvin-Benson cycle, energy supply, electron transport and ROS scavenging (Yin et al., 2019; Suo et al., 2020), indicating that these pathways are critical for chloroplasts in response to salt stress. Furthermore, proteomics also provides new insights into the salt-responsive mechanism in halophytes, for example, proteomics data have revealed that the enhanced chloroplast protein synthesis and processing promote the photosynthetic adaptation to cope with salt stress (Yin et al., 2019). High throughput proteomics can help us acquire more detailed quantitative information about the stress responsive proteins, and will provide further valuable information toward understanding the chloroplast response mechanisms to salt stress. This review tries to collate the sensitivities and adaptive responses of chloroplasts to salt stress, but the adaptive mechanisms are complicated and not completely understood. Many questions remain unclear and need to be further investigated (Isayenkov and Maathuis, 2019), for example, tight regulated ROS can function as retrograde signals for communication between chloroplasts and the nucleus, yet it is still unclear how the chloroplast senses salt stress to initiate the retrograde signaling. The precise details of how salt stress triggers chloroplast response is also not yet clear. In addition, the functions of already identified putative ion transporters in chloroplasts also need further investigation. Answering these questions will help us understand the roles of chloroplasts in plant adaptation to salt stress, and will lay the foundation for genetically breeding more salt-tolerant cultivars.

## Author contributions

XW: Funding acquisition, Writing – original draft, Writing – review & editing. ZC: Writing – review & editing. NS: Conceptualization, Funding acquisition, Writing – review & editing.

## References

[B1] Abdelhamid M. T. Sekara A. Pessarakli M. Alarcón J. J. Brestic M. El-Ramady H.. (2020), 247–268. doi: 10.1007/978-981-15-4120-9_10

[B2] Acosta-Motos J. Ortuño M. Bernal-Vicente A. Diaz-Vivancos P. Sanchez-Blanco M. Hernandez J. (2017). Plant responses to salt stress: Adaptive mechanisms. Agronomy 7, 18. doi: 10.3390/agronomy7010018

[B3] Ahanger M. A. Agarwal R. M. (2017). Salinity stress induced alterations in antioxidant metabolism and nitrogen assimilation in wheat ( Triticum aestivum L) as influenced by potassium supplementation. Plant Physiol. Biochem. 115, 449–460. doi: 10.1016/j.plaphy.2017.04.017 28478373

[B4] Ahmad P. Abass Ahanger M. Nasser AlYemeni M. Wijaya L. Alam P. Ashraf M. (2018). Mitigation of sodium chloride toxicity inSolanum lycopersicumL. by supplementation of jasmonic acid and nitric oxide. J. Plant Interact. 13, 64–72. doi: 10.1080/17429145.2017.1420830

[B5] Akyol T. Y. Yilmaz O. UzİLday B. ÖZgÜR UzİLday R. TÜRkan İ. (2020). Plant response to salinity: an analysis of ROS formation, signaling, and antioxidant defense. Turkish J. Bot. 44, 1–13. doi: 10.3906/bot-1911-15

[B6] Alkhatib R. Abdo N. Mheidat M. (2021). Photosynthetic and Ultrastructural Properties of Eggplant (Solanum melongena) under Salinity Stress. Horticulturae 7, 181. doi: 10.3390/horticulturae7070181

[B7] Allakhverdiev S. I. Nishiyama Y. Miyairi S. Yamamoto H. Inagaki N. Kanesaki Y.. (2002). Salt Stress Inhibits the Repair of Photodamaged Photosystem II by Suppressing the Transcription and Translation ofpsbAGenes inSynechocystis. Plant Physiol. 130, 1443–1453. doi: 10.1104/pp.011114 12428009 PMC166663

[B8] Armbruster U. Carrillo L. R. Venema K. Pavlovic L. Schmidtmann E. Kornfeld A.. (2014). Ion antiport accelerates photosynthetic acclimation in fluctuating light environments. Nat. Commun. 5, 1–8. doi: 10.1038/ncomms6439 PMC4243252 25451040

[B9] Arnon D. I. (1959). Conversion of light into chemical energy in photosynthesis. Nature 184, 10–21. doi: 10.1038/184010a0 13794394

[B10] Asada K. (1999). THE WATER-WATER CYCLE IN CHLOROPLASTS: scavenging of active oxygens and dissipation of excess photons. Annu. Rev. Plant Physiol. Plant Mol. Biol. 50, 601–639. doi: 10.1146/annurev.arplant.50.1.601 15012221

[B11] Ashraf M. Foolad M. R. (2007). Roles of glycine betaine and proline in improving plant abiotic stress resistance. Environ. Exp. Bot. 59, 206–216. doi: 10.1016/j.envexpbot.2005.12.006

[B12] Badawi G. H. Kawano N. Yamauchi Y. Shimada E. Sasaki R. Kubo A.. (2004). Over4).,,.envex of ascorbate peroxidase in tobacco chloroplasts enhances the tolerance to salt stress and water deficit. Physiologia Plantarum 121, 231–238. doi: 10.1111/j.0031-9317.2004.00308.x 15153190

[B13] Barhoumi Z. Djebali W. Chaïbi W. Abdelly C. Smaoui A. (2007). Salt impact on photosynthesis and leaf ultrastructure of Aeluropus littoralis. J. Plant Res. 120, 529–537. doi: 10.1007/s10265-007-0094-z 17534691

[B14] Barkla B. J. Garibay-Hernández A. Melzer M. Rupasinghe T. W. T. Roessner U. (2018). Single cellle.r, analysis of cellular lipid remodelling in response to salinity in the epidermal bladder cells of the model halophyte Mesembryanthemum crystallinum. Plant Cell Environ. 41, 2390–2403. doi: 10.1111/pce.13352 29813189

[B15] Ben Amor N. Jiménez A. Boudabbous M. Sevilla F. Abdelly C. (2020). Chloroplast implication in the tolerance to salinity of the halophyte cakile maritima. Russian J. Plant Physiol. 67, 507–514. doi: 10.1134/S1021443720030048

[B16] Ben Hamed K. Dabbous A. Souid A. Abdelly C. (2020), 1–17. doi: 10.1007/978-3-030-17854-3_48-1

[B17] Bose J. Munns R. Shabala S. Gilliham M. Pogson B. Tyerman S. D. (2017). Chloroplast function and ion regulation in plants growing on saline soils: lessons from halophytes. J. Exp. Bot. 68, 3129–3143. doi: 10.1093/jxb/erx142 28472512

[B18] Bose J. Rodrigo-Moreno A. Shabala S. (2013). ROS homeostasis in halophytes in the context of salinity stress tolerance. J. Exp. Bot. 65, 1241–1257. doi: 10.1093/jxb/ert430 24368505

[B19] Bowyer J. R. Leegood R. C. (1997). “Photosynthesis,” in Plant biochemistry, eds Dey P. M. Harborne J. B. (UK: Academic Press), 49–104. doi: 10.1016/B978-012214674-9/50003-5

[B20] Brownell P. F. (1965). Sodium as an essential micronutrient element for a higher plant (Atriplex vesicaria). Plant Physiol. 40, 460–468. doi: 10.1104/pp.40.3.460 16656111 PMC550317

[B21] Brownell P. F. Wood J. G. (1957). Sodium as an essential micronutrient element for Atriplex vesicaria, Heward. Nature 179, 635–636. doi: 10.1038/179635a0

[B22] Carraretto L. Formentin E. Teardo E. Checchetto V. Tomizioli M. Morosinotto T.. (2013). A thylakoid-located two-pore K+ Channel controls photosynthetic light utilization in plants. Science 342, 114–118. doi: 10.1126/science.1242113 24009357

[B23] Chang L. Guo A. Jin X. Yang Q. Wang D. Sun Y.. (2015). The beta subunit of glyceraldehyde 3-phosphate dehydrogenase is an important factor for maintaining photosynthesis and plant development under salt stress—Based on an integrative analysis of the structural, physiological and proteomic changes in chloroplasts in Thellungiella halophila. Plant Sci. 236, 223–238. doi: 10.1016/j.plantsci.2015.04.010 26025536

[B24] Chaves M. M. Flexas J. Pinheiro C. (2009). Photosynthesis under drought and salt stress: regulation mechanisms from whole plant to cell. Ann. Bot. 103, 551–560. doi: 10.1093/aob/mcn125 18662937 PMC2707345

[B25] Cosentino C. FischernSchliebs E. Bertl A. Thiel G. Homann U. (2010). Na+/H+ antiporters are differentially regulated in response to NaCl stress in leaves and roots of Mesembryanthemum crystallinum. New Phytol. 186, 669–680. doi: 10.1111/j.1469-8137.2010.03208.x 20298477

[B26] Critchley C. (1982). Stimulation of photosynthetic electron transport in a salt-tolerant plant by high chloride concentrations. Nature 298, 483–485. doi: 10.1038/298483a0

[B27] Cui Z. Wang Y. Fan J. Ruan Y. Gao C. Zhang L.. (2007). Research advance of plant osmoregulation. J. Maize Sci. 15, 140–143. doi: 1005-0906 (2007) 06-0140-04

[B28] Davenport R. James R. A. Zakrisson-Plogander A. Tester M. Munns R. (2005). Control of sodium transport in durum wheat. Plant Physiol. 137, 807–818. doi: 10.1104/pp.104.057307 15734907 PMC1065380

[B29] Delatorre-Herrera J. Ruiz K. B. Pinto M. (2021). The importance of non-diffusional factors in determining photosynthesis of two contrasting quinoa ecotypes (Chenopodium quinoa willd.) subjected to salinity conditions. Plants 10, 927. doi: 10.3390/plants10050927 34066627 PMC8148559

[B30] Delfine S. Alvino A. Zacchini M. Loreto F. (1998). Consequences of salt stress on conductance to CO2 diffusion, Rubisco characteristics and anatomy of spinach leaves. Astralian J. Plant Physiol. 25, 395–402. doi: 10.1071/PP97161

[B31] Demetriou G. Neonaki C. Navakoudis E. Kotzabasis K. (2007). Salt stress impact on the molecular structure and function of the photosynthetic apparatus—The protective role of polyamines. Biochim. Biophys. Acta (BBA) - Bioenergetics 1767, 272–280. doi: 10.1016/j.bbabio.2007.02.020 17408588

[B32] Demiral T. Türkan I. (2004). Does exogenous glycinebetaine affect antioxidative system of rice seedlings under NaCl treatment? J. Plant Physiol. 161, 1089–1100. doi: 10.1016/j.jplph.2004.03.009 15535118

[B33] Demmig-Adams B. Adams W. W. (1992). Photoprotection and other responses of plants to high light stress. Annu. Rev. Plant Physiol. Plant Mol. Biol. 43, 599–626. doi: 10.1146/annurev.pp.43.060192.003123

[B34] dos Santos Araújo G. de Oliveira Paula-Marinho S. de Paiva Pinheiro S. K. de Castro Miguel E. de Sousa Lopes L. Camelo Marques E.. (2021). H2O2 priming promotes salt tolerance in maize by protecting chloroplasts ultrastructure and primary metabolites modulation. Plant Sci. 303, 110774. doi: 10.1016/j.plantsci.2020.110774 33487358

[B35] Du Q. Zhao X. H. Xia L. Jiang C. J. Wang X. G. Han Y.. (2019). Effects of potassium deficiency on photosynthesis, chloroplast ultrastructure, ROS, and antioxidant activities in maize (Zea mays L.). J. Integr. Agric. 18, 395–406. doi: 10.1016/S2095-3119(18)61953-7

[B36] Edreva A. (2005). Generation and scavenging of reactive oxygen species in chloroplasts: a submolecular approach. Agriculture Ecosyst. Environ. 106, 119–133. doi: 10.1016/j.agee.2004.10.022

[B37] Evelin H. Giri B. Kapoor R. (2013). Ultrastructural evidence for AMF mediated salt stress mitigation in Trigonella foenum-graecum. Mycorrhiza 23, 71–86. doi: 10.1007/s00572-012-0449-8 22733451

[B38] Ferro M. Brugiere S. Salvi D. Seigneurin-Berny D. Court M. Moyet L.. (2010). AT_CHLORO, a comprehensive chloroplast proteome database with subplastidial localization and curated information on envelope proteins. Mol. Cell. Proteomics 9, 1063–1084. doi: 10.1074/mcp.M900325-MCP200 20061580 PMC2877971

[B39] Fidalgo F. Santos A. Santos I. Salema R. (2004). Effects of longcts,. salt stress on antioxidant defence systems, leaf water relations and chloroplast ultrastructure of potato plants. Ann. Appl. Biol. 145, 185–192. doi: 10.1111/j.1744-7348.2004.tb00374.x

[B40] Finazzi G. Petroutsos D. Tomizioli M. Flori S. Sautron E. Villanova V.. (2015). Ions channels/transporters and chloroplast regulation. Cell Calcium 58, 86–97. doi: 10.1016/j.ceca.2014.10.002 25454594

[B41] Flexas J. Barbour M. M. Brendel O. Cabrera H. M. Carriquí M. Díaz-Espejo A.. (2012). Mesophyll diffusion conductance to CO2: An unappreciated central player in photosynthesis. Plant Sci. 193-194, 70–84. doi: 10.1016/j.plantsci.2012.05.009 22794920

[B42] Flexas J. Bota J. Loreto F. Cornic G. Sharkey T. D. (2004). Diffusive and metabolic limitations to photosynthesis under drought and salinity in C3 plants. Plant Biol. 6, 269–279. doi: 10.1055/s-2004-820867 15143435

[B43] Flowers T. J. Gaur P. M. Gowda C. L. L. Krishnamurthy L. Samineni S. Siddique K. H. M.. (2010). Salt sensitivity in chickpea. Plant Cell Environ. 33, 490–509. doi: 10.1111/j.1365-3040.2009.02051.x 19843257

[B44] Furumoto T. Yamaguchi T. Ohshima-Ichie Y. Nakamura M. Tsuchida-Iwata Y. Shimamura M.. (2011). A plastidial sodium-dependent pyruvate transporter. Nature 476, 472–475. doi: 10.1038/nature10250 21866161

[B45] Gao H.-J. Yang H.-Y. Bai J.-P. Liang X.-Y. Lou Y. Zhang J.-L.. (2015). Ultrastructural and physiological responses of potato (Solanum tuberosum L.) plantlets to gradient saline stress. Front. Plant Sci. 5. doi: 10.3389/fpls.2014.00787 PMC4292236 25628634

[B46] Garg A. K. Kim J.-K. Owens T. G. Ranwala A. P. Choi Y. D. Kochian L. V.. (2002). Trehalose accumulation in rice plants confers high tolerance levels to different abiotic stresses. Proc. Natl. Acad. Sci. 99, 15898–15903. doi: 10.1073/pnas.252637799 12456878 PMC138536

[B47] Gong D. H. Wang G. Z. Si W. T. Zhou Y. Liu Z. Jia J. (2018). Effects of salt stress on photosynthetic pigments and activity of ribulose-1,5-bisphosphate carboxylase/oxygenase in kalidium foliatum. Russian J. Plant Physiol. 65, 98–103. doi: 10.1134/S1021443718010144

[B48] Goussi R. Manaa A. Derbali W. Cantamessa S. Abdelly C. Barbato R. (2018). Comparative analysis of salt stress, duration and intensity, on the chloroplast ultrastructure and photosynthetic apparatus in Thellungiella salsuginea. J. Photochem. Photobiol. B: Biol. 183, 275–287. doi: 10.1016/j.jphotobiol.2018.04.047 29751261

[B49] Grof C. P. L. Johnston M. Brownell P. F. (1989). Effect of sodium nutrition on the ultrastructure of chloroplasts of C4 plants. Plant Physiol. 89, 539–543. doi: 10.1104/pp.89.2.539 16666579 PMC1055878

[B50] Gulzar S. Hussain T. Gul B. Hameed A. (2020). “Photosynthetic adaptations and oxidative stress tolerance in halophytes from warm subtropical region,” in Handbook of Halophytes, eds Grigore, M. N. (Springer, Cham, Switzerland), 1515–1545. doi: 10.1007/978-3-030-57635-6_52

[B51] Hameed A. Ahmed M. Z. Hussain T. Aziz I. Ahmad N. Gul B.. (2021). Effects of salinity stress on chloroplast structure and function. Cells 10, 2023. doi: 10.3390/cells10082023 34440792 PMC8395010

[B52] Hasanuzzaman M. Nahar K. Alam M. M. Bhowmik P. C. Hossain M. A. Rahman M. M.. (2014). Potential use of halophytes to remediate saline soils. BioMed. Res. Int. 2014, 1–12. doi: 10.1155/2014/589341 PMC4109415 25110683

[B53] He W. Yan K. Zhang Y. Bian L. Mei H. Han G. (2021). Contrasting photosynthesis, photoinhibition and oxidative damage in honeysuckle (Lonicera japonica Thunb.) under iso-osmotic salt and drought stresses. Environ. Exp. Bot. 182, 104313. doi: 10.1016/j.envexpbot.2020.104313

[B54] Herdean A. Nziengui H. Zsiros O. Solymosi K. Garab G. Lundin B.. (2016a). The arabidopsis thylakoid chloride channel atCLCe functions in chloride homeostasis and regulation of photosynthetic electron transport. Front. Plant Sci. 7. doi: 10.3389/fpls.2016.00115 PMC4746265 26904077

[B55] Herdean A. Teardo E. Nilsson A. K. Pfeil B. E. Johansson O. N. Ünnep R.. (2016b). A voltage-dependent chloride channel fine-tunes photosynthesis in plants. Nat. Commun. 7, 1–11. doi: 10.1038/ncomms11654 PMC4890181 27216227

[B56] Hernández J. A. Olmos E. Corpas F. J. Sevilla F. del Rio L. A. (1995). Salt-induced oxidative stress in chloroplasts of pea plants. Plant Sci. 105, 151–167. doi: 10.1016/0168-9452(94)04047-8

[B57] Hideg É. Barta C. Kálai T. Vass I. Hideg K. Asada K. (2002). Detection of singlet oxygen and superoxide with fluorescent sensors in leaves under stress by photoinhibition or UV radiation. Plant Cell Physiol. 43, 1154–1164. doi: 10.1093/pcp/pcf145 12407195

[B58] Hiyane R. Hiyane S. Tang A. C. Boyer J. S. (2010). Sucrose feeding reverses shade-induced kernel losses in maize. Ann. Bot. 106, 395–403. doi: 10.1093/aob/mcq132 20616114 PMC2924829

[B59] Hodges M. Dellero Y. Keech O. Betti M. Raghavendra A. S. Sage R.. (2016). Perspectives for a better understanding of the metabolic integration of photorespiration within a complex plant primary metabolism network. J. Exp. Bot. 67, 3015–3026. doi: 10.1093/jxb/erw145 27053720

[B60] Hooper C. M. Tanz S. K. Castleden I. R. Vacher M. A. Small I. D. Millar A. H. (2014). SUBAcon: a consensus algorithm for unifying the subcellular localization data of the Arabidopsis proteome. Bioinformatics 30, 3356–3364. doi: 10.1093/bioinformatics/btu550 25150248

[B61] Hossain M. S. (2019). Present scenario of global salt affected soils, its management and importance of salinity research. Int. Res. J. Biol. Sci. 1, 1–3.

[B62] Hu Y. Schmidhalter U. (2005). Drought and salinity: A comparison of their effects on mineral nutrition of plants. J. Plant Nutr. Soil Sci. 168, 541–549. doi: 10.1002/jpln.200420516

[B63] Huang C. He W. Guo J. Chang X. Su P. Zhang L. (2005). Increased sensitivity to salt stress in an ascorbate-deficient Arabidopsis mutant. J. Exp. Bot. 56, 3041–3049. doi: 10.1093/jxb/eri301 16263910

[B64] Huang L. Li Z. Liu Q. Pu G. Zhang Y. Li J. (2019). Research on the adaptive mechanism of photosynthetic apparatus under salt stress: New directions to increase crop yield in saline soils. Ann. Appl. Biol. 175, 1–17. doi: 10.1111/aab.12510

[B65] Isayenkov S. V. Maathuis F. J. M. (2019). Plant salinity stress: many unanswered questions remain. Front. Plant Sci. 10. doi: 10.3389/fpls.2019.00080 PMC6384275 30828339

[B66] Johnston M. Grof C. P. L. Brownell P. F. (1989). Chlorophyll a/b ratios and photosystem activity of mesophyll and bundle sheath fractions from sodium-deficient C4 plants. Aust. J. Plant Physiol. 16, 449–457. doi: 10.1071/PP9890449

[B67] Kavitha K. George S. Venkataraman G. Parida A. (2010). A salt-inducible chloroplastic monodehydroascorbate reductase from halophyte Avicennia marina confers salt stress tolerance on transgenic plants. Biochimie 92, 1321–1329. doi: 10.1016/j.biochi.2010.06.009 20600571

[B68] Khayyat M. Tehranifar A. Davarynejad G. H. Sayyari-Zahan M. H. (2014). Vegetative growth, compatible solute accumulation, ion partitioning and chlorophyll fluorescence of 'Malas-e-Saveh' and 'Shishe-Kab' pomegranates in response to salinity stress. Photosynthetica 52, 301–312. doi: 10.1007/s11099-014-0034-9

[B69] Khorobrykh S. Havurinne V. Mattila H. Tyystjärvi E. (2020). Oxygen and ROS in photosynthesis. Plants 9, 91. doi: 10.3390/plants9010091 31936893 PMC7020446

[B70] Kleffmann T. Russenberger D. von Zychlinski A. Christopher W. Sjölander K. Gruissem W.. (2004). The arabidopsis thaliana chloroplast proteome reveals pathway abundance and novel protein functions. Curr. Biol. 14, 354–362. doi: 10.1016/j.cub.2004.02.039 15028209

[B71] Kunza H.-H. Gierthb M. Herdeanc A. Satoh-Cruzd M. Kramerd D. M. Speteac C.. (2014). Plastidial transporters KEA1, -2, and -3 are essential for chloroplast osmoregulation, integrity, and pH regulation in Arabidopsis. Proc. Natl. Acad. Sci. United States America 111, 7480–7485. doi: 10.1073/pnas.1323899111 PMC4034250 24794527

[B72] Leisner C. P. Cousins A. B. Offermann S. Okita T. W. Edwards G. E. (2010). The effects of salinity on photosynthesis and growth of the single-cell C4 species Bienertia sinuspersici (Chenopodiaceae). Photosynthesis Res. 106, 201–214. doi: 10.1007/s11120-010-9595-z 20838891

[B73] Lekklar C. Suriya-Arunroj D. Pongpanich M. Comai L. Kositsup B. Chadchawan S.. (2019). Comparative genomic analysis of rice with contrasting photosynthesis and grain production under salt stress. Genes 10, 562. doi: 10.3390/genes10080562 31349693 PMC6722916

[B74] Le Martret B. Poage M. Shiel K. Nugent G. D. Dix P. J. (2011). Tobacco chloroplast transformants expressing genes encoding dehydroascorbate reductase, glutathione reductase, and glutathionerbatransferase, exhibit altered antiredrase, metabolism and improved abiotic stress tolerance. Plant Biotechnol. J. 9, 661–673. doi: 10.1111/j.1467-7652.2011.00611.x 21450042

[B75] Li J.-Y. Yang C. Tian Y.-Y. Liu J.-X. (2022). Regulation of chloroplast development and function at adverse temperatures in plants. Plant Cell Physiol. 63, 580–591. doi: 10.1093/pcp/pcac022 35141744

[B76] Li Q. Yang A. Zhang W.-H. (2017). Comparative studies on tolerance of rice genotypes differing in their tolerance to moderate salt stress. BMC Plant Biol. 17, 1–13. doi: 10.1186/s12870-017-1089-0 28814283 PMC5559854

[B77] Li W. Zhang C. Lu Q. Wen X. Lu C. (2011). The combined effect of salt stress and heat shock on proteome profiling in Suaeda salsa. J. Plant Physiol. 168, 1743–1752. doi: 10.1016/j.jplph.2011.03.018 21663998

[B78] Liu J. Fu C. Li G. Khan M. N. Wu H. (2021). ROS homeostasis and plant salt tolerance: Plant nanobiotechnology updates. Sustainability 13, 3552. doi: 10.3390/su13063552

[B79] Liu L. Wang B. (2021). Protection of halophytes and their uses for cultivation of saline-alkali soil in China. Biology 10, 353. doi: 10.3390/biology10050353 33922035 PMC8143469

[B80] Lodeyro A. F. Giró M. Poli H. O. Bettucci G. Cortadi A. Ferri A. M.. (2016). Suppression of reactive oxygen species accumulation in chloroplasts prevents leaf damage but not growth arrest in salt-stressed tobacco plants. PLoS One 11, e0159588. doi: 10.1371/journal.pone.0159588 27441560 PMC4956149

[B81] Loreto F. Centritto M. Chartzoulakis K. (2003). Photosynthetic limitations in olive cultivars with different sensitivity to salt stress. Plant Cell Environ. 26, 595–601. doi: 10.1046/j.1365-3040.2003.00994.x

[B82] Lu C. Li L. Liu X. Chen M. Wan S. Li G. (2023). Salt stress inhibits photosynthesis and destroys chloroplast structure by downregulating chloroplast development–related genes in robinia pseudoacacia seedlings. Plants 12, 1283. doi: 10.3390/plants12061283 36986971 PMC10054032

[B83] Luo X. Wu J. Li Y. Nan Z. Guo X. Wang Y.. (2013). Synergistic effects of ghSOD1 and ghCAT1 overexpression in cotton chloroplasts on enhancing tolerance to methyl viologen and salt stresses. PLoS One 8, e54002. doi: 10.1371/journal.pone.0054002.g001 23335985 PMC3545958

[B84] MaChado R. Serralheiro R. (2017). Soil salinity: Effect on vegetable crop growth. Management practices to prevent and mitigate soil salinization. Horticulturae 3, 30. doi: 10.3390/horticulturae3020030

[B85] Manaa A. Goussi R. Derbali W. Cantamessa S. Abdelly C. Barbato R. (2019). Salinity tolerance of quinoa (Chenopodium quinoa Willd) as assessed by chloroplast ultrastructure and photosynthetic performance. Environ. Exp. Bot. 162, 103–114. doi: 10.1016/j.envexpbot.2019.02.012

[B86] Maxwell K. Johnson G. N. (2000). Chlorophyll fluorescence—a practical guide. J. Exp. Bot. 51, 659–668. doi: 10.1093/jexbot/51.345.659 10938857

[B87] Mittler R. (2002). Oxidative stress, antioxidants and stress tolerance. Trends Plant Sci. 7, 405–410. doi: 10.1016/S1360-1385(02)02312-9 12234732

[B88] Mittler R. Vanderauwera S. Gollery M. Van Breusegem F. (2004). Reactive oxygen gene network of plants. Trends Plant Sci. 9, 490–498. doi: 10.1016/j.tplants.2004.08.009 15465684

[B89] Müller M. Kunz H. H. Schroeder J. I. Kemp G. Young H. S. Neuhaus H. E. (2014). Decreased capacity for sodium export out of Arabidopsis chloroplasts impairs salt tolerance, photosynthesis and plant performance. Plant J. 78, 646–658. doi: 10.1111/tpj.12501 24617758

[B90] Munns R. Passioura J. B. Colmer T. D. Byrt C. S. (2019). Osmotic adjustment and energy limitations to plant growth in saline soil. New Phytol. 225, 1091–1096. doi: 10.1111/nph.15862 31006123

[B91] Munns R. Tester M. (2008). Mechanisms of salinity tolerance. Annu. Rev. Plant Biol. 59, 651–681. doi: 10.1146/annurev.arplant.59.032607.092911 18444910

[B92] Murata N. Takahashi S. Nishiyama Y. Allakhverdiev S. I. (2007). Photoinhibition of photosystem II under environmental stress. Biochim. Biophys. Acta (BBA) - Bioenergetics 1767, 414–421. doi: 10.1016/j.bbabio.2006.11.019 17207454

[B93] Neuhaus H. E. Emes M. J. (2000). Nonphotosynthetic metabolism in plastids. Annu. Rev. Plant Physiol. Plant Mol. Biol. 51, 111–140. doi: 10.1146/annurev.arplant.51.1.111 15012188

[B94] Nishiyama Y. Yamamoto H. Allakhverdiev S. I. Inaba M. Yokota A. Murata N. (2001). Oxidative stress inhibits the repair of photodamage to the photosynthetic machinery. EMBO J. 20, 5587–5594. doi: 10.1093/emboj/20.20.5587 11598002 PMC125664

[B95] Niyogi K. K. (1999). PHOTOPROTECTION REVISITED: genetic and molecular approaches. Annu. Rev. Plant Physiol. Plant Mol. Biol. 50, 333–359. doi: 10.1146/annurev.arplant.50.1.333 15012213

[B96] Nouri M.-Z. Moumeni A. Komatsu S. (2015). Abiotic stresses: Insight into gene regulation and protein expression in photosynthetic pathways of plants. Int. J. Mol. Sci. 16, 20392–20416. doi: 10.3390/ijms160920392 26343644 PMC4613210

[B97] Ozgur R. Uzilday B. Sekmen A. H. Turkan I. (2013). Reactive oxygen species regulation and antioxidant defence in halophytes. Funct. Plant Biol. 40, 832. doi: 10.1071/FP12389 32481154

[B98] Papageorgiou G. C. Murata N. (1995). The unusually strong stabilizing effects of glycine betaine on the structure and function of the oxygen-evolving Photosystem II complex. Photosynthesis Res. 44, 243–252. doi: 10.1007/BF00048597 24307094

[B99] Parida A. K. Das A. B. (2005). Salt tolerance and salinity effects on plants: a review. Ecotoxicology Environ. Saf. 60, 324–349. doi: 10.1016/j.ecoenv.2004.06.010 15590011

[B100] Park E. J. JekniĆ Z. Pino M. T. Murata N. Chen T. H. H. (2007). Glycinebetaine accumulation is more effective in chloroplasts than in the cytosol for protecting transgenic tomato plants against abiotic stress. Plant Cell Environ. 30, 994–1005. doi: 10.1111/j.1365-3040.2007.01694.x 17617827

[B101] Planchet E. Verdu I. Delahaie J. Cukier C. Girard C. Morère-Le Paven M.-C.. (2014). Abscisic acid-induced nitric oxide and proline accumulation in independent pathways under water-deficit stress during seedling establishment in Medicago truncatula. J. Exp. Bot. 65, 2161–2170. doi: 10.1093/jxb/eru088 24604737

[B102] Qiu N. Lu Q. Lu C. (2003). Photosynthesis, photosystem II efficiency and the xanthophyll cycle in the salteophylls halophyte Atriplex centralasiatica. New Phytol. 159, 479–486. doi: 10.1046/j.1469-8137.2003.00825.x 33873362

[B103] Queirós F. Rodrigues J. A. Almeida J. M. Almeida D. P. F. Fidalgo F. (2011). Differential responses of the antioxidant defence system and ultrastructure in a salt-adapted potato cell line. Plant Physiol. Biochem. 49, 1410–1419. doi: 10.1016/j.plaphy.2011.09.020 22078378

[B104] Raghavendra A. S. Padmasree K. (2003). Beneficial interactions of mitochondrial metabolism with photosynthetic carbon assimilation. Trends Plant Sci. 8, 546–553. doi: 10.1016/j.tplants.2003.09.015 14607100

[B105] Robinson S. P. Downton W. J. S. (1984). Potassium, chloroplasts sodium, and chloride content of isolated in relation to ionic compartmentation. Arch. Biochem. Biophysics 228, 197–206. doi: 10.1016/0003-9861(84)90061-4 6696431

[B106] Robinson S. P. Downton W. J. S. (1985). Potassium, sodium and chloride ion concentrations in leaves and isolated chloroplasts of the halophyte Suaeda Australis R. Br. Aust. J. Plant Physiol. 12, 471–479. doi: 10.1071/PP9850471

[B107] Salama S. Trivedi S. Busheva M. Arafa A. A. Garab G. Erdei L. (1994). Effects of naCl salinity on growth, cation accumulation, chloroplast structure and function in wheat cultivars differing in salt tolerance. J. Plant Physiol. 144, 241–247. doi: 10.1016/S0176-1617(11)80550-X

[B108] Sevilla F. Camejo D. Ortiz-Espín A. Calderón A. Lázaro J. J. Jiménez A. (2015). The thioredoxin/peroxiredoxin/sulfiredoxin system: current overview on its redox function in plants and regulation by reactive oxygen and nitrogen species. J. Exp. Bot. 66, 2945–2955. doi: 10.1093/jxb/erv146 25873657

[B109] Shen B. Jensen R. C. Bohner H. (1997a). Mannitol protects against oxidation by hydroxyl radicals. Plant Physiol. 115, 527–532. doi: 10.1104/pp.115.2.527 12223821 PMC158511

[B110] Shen B. Jensen R. G. Bohnert H. J. (1997b). lncreased resistance to oxidative stress in transgenic plants by targeting mannitol biosynthesis to chloroplasts. Plant Physiol. 113, 1177–1183. doi: 10.1104/pp.113.4.1177 9112772 PMC158240

[B111] Shu S. Yuan Y. Chen J. Sun J. Zhang W. Tang Y.. (2015). The role of putrescine in the regulation of proteins and fatty acids of thylakoid membranes under salt stress. Sci. Rep. 5, 1–16. doi: 10.1038/srep14390 PMC4593046 26435404

[B112] Singh A. Roychoudhury A. (2021). Gene regulation at transcriptional and postscriptional90..e levels to combat salt stress in plants. Physiologia Plantarum 173, 1556–1572. doi: 10.1111/ppl.13502 34260753

[B113] Song Y. Feng L. Alyafei M. A. M. Jaleel A. Ren M. (2021). Function of chloroplasts in plant stress responses. Int. J. Mol. Sci. 22, 13464. doi: 10.3390/ijms222413464 34948261 PMC8705820

[B114] Štefanić P. P. Koffler T. Adler G. Bar-Zvi D. (2013). Chloroplasts of salt-grown arabidopsis seedlings are impaired in structure, genome copy number and transcript levels. PLoS One 8, e82548. doi: 10.1371/journal.pone.0082548 24340039 PMC3855474

[B115] Suo J. Zhang H. Zhao Q. Zhang N. Zhang Y. Li Y.. (2020). Na2CO3-responsive photosynthetic and ROS scavenging mechanisms in chloroplasts of alkaligrass revealed by phosphoproteomics. Genomics Proteomics Bioinf. 18, 271–288. doi: 10.1016/j.gpb.2018.10.011 PMC7801222 32683046

[B116] Suo J. Zhao Q. David L. Chen S. Dai S. (2017). Salinity response in chloroplasts: Insights from gene characterization. Int. J. Mol. Sci. 18, 1011. doi: 10.3390/ijms18051011 28481319 PMC5454924

[B117] Suzuki N. Koussevitzky S. Mittler R. O. N. Miller G. A. D. (2012). ROS and redox signalling in the response of plants to abiotic stress. Plant Cell Environ. 35, 259–270. doi: 10.1111/j.1365-3040.2011.02336.x 21486305

[B118] Szabados L. Savouré A. (2010). Proline: a multifunctional amino acid. Trends Plant Sci. 15, 89–97. doi: 10.1016/j.tplants.2009.11.009 20036181

[B119] Takahashi S. Murata N. (2008). How do environmental stresses accelerate photoinhibition? Trends Plant Sci. 13, 178–182. doi: 10.1016/j.tplants.2008.01.005 18328775

[B120] Teakle N. L. Tyerman S. D. (2010). Mechanisms of Clc transport contributing to salt tolerance. Plant Cell Environ. 33, 566–589. doi: 10.1111/j.1365-3040.2009.02060.x 19895402

[B121] Tian F. Wang W. Liang C. Wang X. Wang G. Wang W. (2017). Overaccumulation of glycine betaine makes the function of the thylakoid membrane better in wheat under salt stress. Crop J. 5, 73–82. doi: 10.1016/j.cj.2016.05.008

[B122] Tränkner M. Tavakol E. Jákli B. (2018). Functioning of potassium and magnesium in photosynthesis, photosynthate translocation and photoprotection. Physiologia Plantarum 163, 414–431. doi: 10.1111/ppl.12747 29667201

[B123] Tseng M. J. Liu C.-W. Yiu J.-C. (2007). Enhanced tolerance to sulfur dioxide and salt stress of transgenic Chinese cabbage plants expressing both superoxide dismutase and catalase in chloroplasts. Plant Physiol. Biochem. 45, 822–833. doi: 10.1016/j.plaphy.2007.07.011 17851086

[B124] Uzilday B. Ozgur R. Sekmen A. H. Yildiztugay E. Turkan I. (2014). Changes in the alternative electron sinks and antioxidant defence in chloroplasts of the extreme halophyte Eutrema parvulum (Thellungiella parvula) under salinity. Ann. Bot. 115, 449–463. doi: 10.1093/aob/mcu184 25231894 PMC4332603

[B125] van Wijk K. J. (2000). Proteomics of the chloroplast: experimentation and prediction. Trends Plant Sci. 5, 420–425. doi: 10.1016/S1360-1385(00)01737-4 11044718

[B126] Vineeth T. V. Krishna G. K. Pandesha P. H. Sathee L. Thomas S. James D.. (2023). Photosynthetic machinery under salinity stress: Trepidations and adaptive mechanisms. Photosynthetica 61, 73–93. doi: 10.32615/ps.2023.002 PMC11515832 39650121

[B127] Wiciarz M. Gubernator B. Kruk J. Niewiadomska E. (2015). Enhanced chloroplastic generation of H2O2 in stresstiontic023 Thellungiella salsuginea in comparison to Arabidopsis thaliana. Physiologia Plantarum 153, 467–476. doi: 10.1111/ppl.12248 24961163 PMC4359041

[B128] Wu T.-M. Lin W.-R. Kao C. H. Hong C.-Y. (2015). Gene knockout of glutathione reductase 3 results in increased sensitivity to salt stress in rice. Plant Mol. Biol. 87, 555–564. doi: 10.1007/s11103-015-0290-5 25636203

[B129] Wu T.-M. Lin W.-R. Kao Y.-T. Hsu Y.-T. Yeh C.-H. Hong C.-Y.. (2013). Identification and characterization of a novel chloroplast/mitochondria co-localized glutathione reductase 3 involved in salt stress response in rice. Plant Mol. Biol. 83, 379–390. doi: 10.1007/s11103-013-0095-3 23783412

[B130] Wu J. Zhang J. Li X. Xu J. Wang L. (2016). Identification and characterization of a PutCu/Zn-SOD gene from Puccinellia tenuiflora (Turcz.) Scribn. et Merr. Plant Growth Regul. 79, 55–64. doi: 10.1007/s10725-015-0110-6

[B131] Wutipraditkul N. Wongwean P. Buaboocha T. (2015). Alleviation of salt-induced oxidative stress in rice seedlings by proline and/or glycinebetaine. Biol. plantarum 59, 547–553. doi: 10.1007/s10535-015-0523-0

[B132] Xiong J. Sun Y. Yang Q. Tian H. Zhang H. Liu Y.. (2017). Proteomic analysis of early salt stress responsive proteins in alfalfa roots and shoots. Proteome Sci. 15, 1–19. doi: 10.1186/s12953-017-0127-z 29093645 PMC5663070

[B133] Yan K. Shao H. Shao C. Chen P. Zhao S. Brestic M.. (2013). Physiological adaptive mechanisms of plants grown in saline soil and implications for sustainable saline agriculture in coastal zone. Acta Physiologiae Plantarum 35, 2867–2878. doi: 10.1007/s11738-013-1325-7

[B134] Yang Z. Li J.-L. Liu L.-N. Xie Q. Sui N. (2020). Photosynthetic regulation under salt stress and salt-tolerance mechanism of sweet sorghum. Front. Plant Sci. 10. doi: 10.3389/fpls.2019.01722 PMC6974683 32010174

[B135] Yang W.-J. Rich P. J. Axtell J. D. Wood K. V. Bonham C. C. Ejeta G.. (2003). Genotypic variation for glycinebetaine in sorghum. Crop Sci. 43, 162–169. doi: 10.2135/cropsci2003.1620

[B136] Yin Z. Zhang H. Zhao Q. Yoo M.-J. Zhu N. Yu J.. (2019). Physiological and comparative proteomic analyses of saline-alkali NaHCO3-responses in leaves of halophyte Puccinellia tenuiflora. Plant Soil 437, 137–158. doi: 10.1007/s11104-019-03955-9

[B137] Yu J. Li Y. Qin Z. Guo S. Li Y. Miao Y.. (2020). Plant chloroplast stress response: Insights from thiol redox proteomics. Antioxidants Redox Signaling 33, 35–57. doi: 10.1089/ars.2019.7823 31989831

[B138] Zahra N. Al Hinai M. S. Hafeez M. B. Rehman A. Wahid A. Siddique K. H. M.. (2022). Regulation of photosynthesis under salt stress and associated tolerance mechanisms. Plant Physiol. Biochem. 178, 55–69. doi: 10.1016/j.plaphy.2022.03.003 35276596

[B139] Zhai C.-Z. Zhao L. Yin L.-J. Chen M. Wang Q.-Y. Li L.-C.. (2013). Two wheat glutathione peroxidase genes whose products are located in chloroplasts improve salt and H2O2 tolerances in arabidopsis. PLoS One 8, e73989. doi: 10.1371/journal.pone.0073989.g001 24098330 PMC3788784

[B140] Zhang Y. Kaiser E. Marcelis L. F. M. Yang Q. Li T. (2020). Salt stress and fluctuating light have separate effects on photosynthetic acclimation, but interactively affect biomass. Plant Cell Environ. 43, 2192–2206. doi: 10.1111/pce.13810 32463133

[B141] Zhang Q. Y. Wang L. Y. Kong F. Y. Deng Y. S. Li B. Meng Q. W. (2012). Constitutive accumulation of zeaxanthin in tomato alleviates salt stressatesonna photoinhibition and photooxidation. Physiologia Plantarum 146, 363–373. doi: 10.1111/j.1399-3054.2012.01645.x 22578286

[B142] Zhao Y. Ai X. Wang M. Xiao L. Xia G. (2016). A putative pyruvate transporter TaBASS2 positively regulates salinity tolerance in wheat via modulation of ABI4 expression. BMC Plant Biol. 16, 1–12. doi: 10.1186/s12870-016-0795-3 27160076 PMC4862123

[B143] Zhao K. Fan H. Ungar I. A. (2002). Survey of halophyte species in China. Plant Sci. 163, 491–498. doi: 10.1016/S0168-9452(02)00160-7

[B144] Zheng X. Tan D. X. Allan A. C. Zuo B. Zhao Y. Reiter R. J.. (2017). Chloroplastic biosynthesis of melatonin and its involvement in protection of plants from salt stress. Sci. Rep. 7, 1–12. doi: 10.1038/srep41236 28145449 PMC5286529

